# Low Fat Yoghurts Produced with Different Protein Levels and Alternative Natural Sweeteners

**DOI:** 10.3390/foods13020250

**Published:** 2024-01-12

**Authors:** Lara Campos, Paulina Tuma, Tânia Silva, David Gomes, Carlos Dias Pereira, Marta H. F. Henriques

**Affiliations:** 1 Research Centre for Natural Resources Environment and Society (CERNAS), Coimbra Agriculture School, Bencanta, 3045-601 Coimbra, Portugal; lara.campos@esac.pt (L.C.); david@esac.pt (D.G.); cpereira@esac.pt (C.D.P.); 2 Department of Food Technology and Human Nutrition, West Pomeranian University of Technology, al. Piastów 42, 71-065 Szczecin, Poland; paulina272@gmail.com; 3 Polytechnic Institute of Coimbra, Coimbra Agriculture School, Bencanta, 3045-601 Coimbra, Portugal; tania.silva@esac.pt

**Keywords:** agave syrup, honey, natural sweeteners, protein content, stevia, xylitol, yoghurts

## Abstract

The food industry is looking for substitutes for sucrose in food items due to the excessive consumption of products with added sugar and the demand for healthier products. Alternative natural sweeteners can help achieve this goal. Different types of low-fat yoghurts (1% fat), with low-protein and high-protein levels (3% and 4.5–6.5% protein, respectively), were produced using alternative natural sweeteners. The low-protein yoghurts were made with stevia (0.03% *w*/*w*) or agave syrup (4.5% *w*/*w*). The high-protein yoghurts were made with stevia (0.04% *w*/*w*), xylitol (6% *w*/*w*) or honey (6% *w*/*w*). Sucrose (6% *w*/*w*) was used as a control in both trials. pH and titratable acidity, CIEL*a*b* color parameters, syneresis index, rheology and the texture profile of the low-fat yoghurts were evaluated over refrigerated storage. All products underwent sensory evaluation by an untrained panel. The high-protein yoghurts were found to be more acidic (>1% as lactic acid), had a lower syneresis index (between 2.1 and 16.2%) and a better consistency (stronger gel structure) than the low-protein yoghurts. In terms of rheological parameters, stevia-sweetened yoghurts scored higher than the other sweetened yoghurts, showing a better gel structure. The different sweeteners tested did not significantly affect the sensory properties of the yoghurts, although the high-protein yoghurts scored higher for most of the attributes evaluated. Overall, consumers preferred stevia-sweetened yoghurts to yoghurts sweetened with sucrose or agave for the low-protein yoghurts. Of the tested formulations, those containing high protein with the alternative natural sweetener xylitol received higher scores in all attributes. These results reveal the potential of the tested natural sweeteners as sucrose substitutes, while contributing to improving the nutritional value of yoghurts.

## 1. Introduction

Nowadays, yoghurt is one of the most important dairy products, being consumed worldwide since it has many health benefits, is affordable and has a wide range of flavors and forms [1,2]. Yoghurt is a fermented dairy product, most commonly manufactured from cow’s milk. Milk proteins are coagulated by lactic acid produced by the typical microorganisms *Streptococcus thermophilus* and *Lactobacillus delbrueckii* subsp. *bulgaricus*, and a gel structure is formed [3,4]. Probiotic bacteria, prebiotics, vitamins, minerals, aromas, fruits, and sweeteners, can be also added to yoghurts [5]. In several countries, the addition of bacteria other than those mentioned above, *S. thermophilus* and *L. bulgaricus*, means that the product can no longer be designated as yoghurt, being allowed only the designation of fermented dairy product. 

Sweetened yoghurts can contain more than 8% of added sugar [2,6]. Sugar is an important component in dairy products [7], especially yoghurts, since consumers consider the sweet flavor as one of the most significant sensory characteristics of this product [8].

Regarding functional properties, during yoghurt production, sugar acts as a bulking agent, affects physical properties such as texture, viscosity and color, can work as a preservative and changes the flavor perception [9]. The main source of sugar used to promote sweetness in the food industry is sucrose [7,10]. However, it is well-known that excessive sugar intake can have a negative effect on the consumer’s health, resulting in obesity, diabetes, tooth decay, cardiovascular disease, hypertriglyceridemia and kidney disease [7]. These negative effects may contribute to people’s increased awareness and demand for healthier products, especially with low, or no added, sugars [11]. Some studies have shown that when sucrose is reduced or replaced by other artificial or natural sweeteners, the properties, quality and acceptability of yoghurts can be influenced [2,7].

Currently, artificial sweeteners such as saccharin, aspartame and sucralose, which are non-nutritive, are the most used substitutes for sucrose [10,12]. However, these products are also associated with the specific health problems caused by sucrose [10,12]. As a result, the market for natural and healthy products is growing and dairy products with natural sweeteners are developing fast [2,10,12]. Some of the natural sweeteners that have been tested as alternatives to sucrose and artificial sweeteners are xylitol, stevia and honey [11]. Agave syrup can also be used as a substitute for sucrose. However, very few studies have been conducted with this objective [13].

Xylitol can be extracted from fruits or vegetables; however, its yield makes this process unprofitable [14]. Thus, the most effective way to obtain xylitol is through the production of xylose or hemicellulose hydrolysates using bacteria, fungi or yeasts [10,15]. Xylitol has no unpleasant after taste and its sweetness is equivalent to sucrose, but unlike sucrose, it is anticariogenic and can be consumed by diabetics [15].

Stevia is the common name for stevioside, a physiochemically stable component extracted from the leaves of *Stevia rebaudiana* Bertoni. Stevioside is the main steviol glycoside (90% of total glycosides) and is 300 times sweeter than sucrose [10,11,12,16]. This component has therapeutic effects such as antihypertensive, anti-inflammatory, antitumor, antidiarrheal, diuretic and antihyperglycemic [12].

Honey is a natural syrup deposited by bees in honeycombs, containing mainly fructose (40–50%) and glucose (32–37%) [17]. Although honey has a low pH that makes it compatible with many food products and has a wide range of beneficial nutritional properties, it is uncommonly used as a sweetener in yoghurts [3]. Varga et al. [3] tested honey as a sweetener in yoghurts and found that the pH, lactic acid levels and the viability of characteristic microorganisms in yoghurts were not altered. It was also concluded that, according to the concentration, honey can contribute to improving the sensory characteristics of the final product.

As a natural sweetener, agave is obtained primarily from *Agave tequilana* Weber. It consists of fructans, which are considered prebiotic dietary fibers [18]. In yoghurts, agave has been tested as a fat substitute. Sensory evaluation concluded that agave did not affect sensory attributes (taste, smell and color) and improved mouthfeel and texture [13]. Agave was used as a sweetener in muffins [19] and chocolate [20]. In muffins, the best sensory properties were achieved with the replacement of 75% of sucrose by agave [19]. In chocolate, samples containing agave had the highest rating scores regarding the sensory attributes [20]. 

The food industry encounters various challenges when attempting to substitute sugar in food products due to its impact on physicochemical properties, product quality, sensory perception, and consumer acceptance. Moreover, the growing trend towards consuming healthier low-fat foods negatively affects the texture/creaminess of yoghurts. One potential solution to mitigate this issue is to increase its the protein content [6,21].

According to the literature review it was observed that the main applications and studies with natural sweeteners have been focused on foods such as bakery products, beverages or snack bars. It was also found a lack of information about the influence of the use of natural sweeteners in dairy products, and even less studies have been conducted to infer the physicochemical and sensorial changes in yoghurts, especially in those classified as low-fat or high-protein products.

Therefore, to assess the potential of different natural sweeteners on the physicochemical and sensory properties of yoghurt, different types of low-fat (1% fat), low-protein and high-protein yoghurts (3% and 4.5–6.5% protein, respectively), have been produced with alternative natural sweeteners such as stevia (0.03% and 0.04% *w*/*w*), agave syrup (4.5% *w*/*w*), xylitol (6% *w*/*w*) and honey (6% *w*/*w*) and compared with sucrose (6% *w*/*w*) as a control.

## 2. Materials and Methods

Low-fat sweet yoghurts were produced with both low- and high-protein content. To increase the protein content, skimmed milk powder, which is a more cost-effective ingredient than whey protein concentrates or caseinate powder, was used. The skimmed milk powder purchased from Lactogal (Portugal) has a composition of 36% (*w*/*w*) of protein, 53% (*w*/*w*) of carbohydrates, 0.9% (*w*/*w*) of fat and 1% (*w*/*w*) of salts. Stevia and xylitol were purchased from BioSamara (BioSamara Iberia—Showroom, atendimento e Armazém, Colares, Portugal), agave was purchased from NatureFoods (Dietimpore, Lisboa, Portugal), honey was purchased from SerraMel (Euromel, Lda—Apicultores, Penamacor, Portugal), and sucrose from RAR—Refinarias de Açúcar Reunidas, S.A. (Porto, Portugal). Yoghurts were initially characterized in terms of moisture, ashes, protein, fat and carbohydrate content. The pH, total acidity, color (L*, a*, b*), texture and rheological parameters, as well as syneresis, were evaluated weekly. On the 7th day, a sensory analysis was performed to understand products’ acceptability.

### 2.1. Yoghurts Production

Whole bovine milk was supplied by a local milk producer, skimmed in a Westfalia™ separator type ADB (GEA Group, Oelde, Germany) to standardize the fat content to 1% (*w*/*w*), batch pasteurized at 91–92 °C for 25–30 s and slowly cooled in a refrigeration chamber. This slow cooling process facilitates the denaturation and aggregation of whey protein to caseins. After cooling to 65 °C, pasteurized skimmed milk (35 L) was divided into two portions to produce: (i) 15 L of low-protein yoghurts and (ii) 20 L of high-protein yoghurts supplemented with skimmed milk powder to test the influence of increasing total solids on the texture, rheology and sensory perception of yoghurt produced with natural sweeteners. For low-protein yoghurts, three variants were produced: with sucrose (6%, *w*/*w*) as the control, with stevia (0.03%, *w*/*w*) and with agave (4.5%, *w*/*w*). For the high-protein yoghurts, four variants were made: with sucrose (6%, *w*/*w*) as the control, with stevia (0.04%, *w*/*w*), with xylitol (6%, *w*/*w*) and with honey (6%, *w*/*w*). The quantities of natural sweeteners employed in yoghurt formulations were determined based on their respective sweetness levels compared to sucrose. Since xylitol and honey have similar sweetness to sucrose, the same concentration was used. The sweetness of agave syrup is often considered to be around 1.5 times that of sucrose. Therefore, a concentration of 4.5% was chosen. Regarding stevia, its concentration was significantly lower due to its 300-times higher sweetening value. The slightly different amounts of stevia on low-protein and high-protein yoghurts (0.03 and 0.04% *w*/*w*, respectively) were chosen to avoid differences in the perceived sweetness that could result from the higher solids content of high-protein yoghurts.

All the ingredients for each formulation were added to the milk after cooling to 65 °C and the mixture was homogenized at 20 MPa. Before filling and packaging, the mixture was stirred for 10 min at 43 °C and inoculated with 0.005% (*w*/*w*) of a mixed culture of *Streptococcus thermophilus* and *Lactobacillus bulgaricus* (Ezal YO-MIX 601). The fermentation step was performed in 50 mL polystyrene cups at a constant temperature of 43 ± 1 °C until the yoghurt’s pH reached 4.6 ± 0.1, which occurred in 4 h. The yoghurts were then stored at 4 ± 2 °C. After one day of cold storage, the biochemical composition and functional properties of some yoghurt samples were evaluated, while the remaining samples were evaluated on days 7, 14 and 21.

### 2.2. Compositional Analysis

Total solids were determined by drying the samples in a Schutzart DIN 40050-IP20 Memmert™ oven (Schwabach, Germany), according to NP 703:1982 for yoghurts [22].

The ash content was determined by the incineration of dry samples in a Nabertherm™ model LE 4/11/R6 electric muffle furnace (Bremen, Germany) at 550 °C for 4 h, according to AOAC method [23].

The fat content was determined according to NP 469:2002 for milk [24]. The total nitrogen content was determined by the Kjeldahl method in the Digestion System 6 1007 Digester Tecator™ (Foss Analytical, Häganäs, Sweden) following the AOAC method, and the conversion factor of 6.38 was used to calculate the percentage of protein [23].

Carbohydrates were determined by the difference between total solids and the sum of ash, fat and protein, all expressed in percentage (% *w*/*w*), according to Equation (1).

(1) %Carbohydrates=%Total Solids−(%Ash+%Fat+%Protein)

Total solids, ash content and fat analysis were performed in triplicate. Protein and carbohydrates evaluation were performed in duplicate.

### 2.3. pH and Titratable Acidity

The pH was determined with a HI 9025 pH meter (Hanna Instruments, Leighton Buzzard, UK) in order to monitor its evolution immediately after the production of the fermented products and during storage. The pH meter was previously calibrated with 7.01 (HI5007) and 4.01 (HI5004) Hanna buffer solutions.

The titratable acidity, expressed in the percentage of lactic acid (*w*/*w*), was determined by means of titration using a 0.1 N NaOH solution according to the technique described in NP 701:1982 for yoghurts [25]. Triplicates were performed for both parameters.

### 2.4. Color Analysis

The color was determined with a Minolta Chroma Meter, model CR-200B colorimeter (Konica Minolta, Tokyo, Japan) calibrated with a white standard (CR-A47, L*standard = 97.03; a*standard = −0.67; b*standard = 5.57). The following conditions were used: illuminant C, 1 cm diameter aperture and 10° standard observer. The color coordinates (L*, a* and b*) were measured in the CIEL*a*b* system. Six measurements were taken for each sample on the yoghurt surface.

### 2.5. Syneresis Index

Yoghurt samples were centrifuged at 350 rpm in a refrigerated centrifuge Hettich Rotanta 460R model (Andreas Hettich GmbH & Co. KG, Tuttlingen, Germany) for 10 min at 5 °C. The supernatant was collected and weighed. Syneresis was reported as the weight of the supernatant relative to the weight of the yoghurt. The water holding capacity (%) was reported as the held water values calculated as the ratio of the weight of water retained in the yoghurts after centrifugation [26].

### 2.6. Rheological Analysis

The rheological properties of the different samples were determined in a HAAKE RheoStress 6000 rheometer (ThermoHaake™, Thermo Scientific, Waltham, MA, USA) equipped with a Peltier plate for temperature control in oscillatory mode. This analysis was performed on the 7th, 14th and 21st day of refrigerated storage. The measurement system consisted of a plate and plate geometry P35 TiL (diameter of 35 mm). Stress sweep tests were conducted at 1 Hz to determine the linear viscoelastic range of the yoghurts. Each yoghurt was transferred onto the rheometer plate at 5 °C and the excess material was wiped off with a spatula. The rheological properties, elastic modulus (G′), viscous modulus (G″), complex viscosity (η*) and the damping factor (tan δ) were evaluated in the range of 0.05–1.00 Hz at 3 Pa. Values of G′, G″, η* and tan δ were recorded at 1 Hz for comparison.

The HAAKE RheoWin software version 4.86.0002 (Thermo Fisher Scientific, Waltham, MA, USA) was employed for data evaluation. Three measurements were taken for each sample.

From the obtained data it was possible to apply the power law model equation, according to Equation (2), and determine the *a* and *b* parameters.

(2) $$G^{\prime }=a\times \omega^{b}$$

where G′ is the elastic modulus, *a* is the consistency index, ω is the frequency (Hz) and *b* is the slope of the curve.

### 2.7. Texture Analysis

A Stable Micro Systems texture analyzer, model TA.XT Express Enhanced (Godalming, UK), was used to evaluate the texture parameters (hardness, adhesiveness, springiness, gumminess, cohesiveness and resilience) of yoghurts 1, 7, 14 and 21 days after production, and the results were calculated using the Specific Expression PC software (version 1,1,9,0). Six replicates were made for each tested sample.

### 2.8. Sensory Analysis

The sensorial properties of yoghurts were evaluated after 7 days of refrigerated storage, by 33 non-trained individuals. This period was chosen as a reasonable time frame for the product’s distribution cycle, from production to consumer. Panelists were asked to evaluate flavor, taste, consistency and appearance on a 9-point hedonic scale (where 1 = very unsatisfied and 9 = very satisfied), using yoghurt samples that were placed on individual plates coded using a random tree-digit-code. Prior to sensory tests, panelists were informed about the objectives of the work and firmed an informed consent form regarding their participation in the test.

### 2.9. Statistical Analysis

All data are expressed as mean values ± standard deviation. Statistical analysis was performed using GraphPad Prims Software version 8.0.2 (GraphPad Software, Inc., San Diego, CA, USA). A one-way analysis of variance (ANOVA) using Tukey’s test was performed to determine the differences between the means obtained in compositional and sensorial analysis at significance level of 5%.

For pH, titratable acidity, color parameters, syneresis index, rheology and texture parameters, a two-way analysis of variance (ANOVA) using Tukey’s test was performed to determine the differences between the means at significance level of 5%.

## 3. Results and Discussion

The chemical composition of the low-fat yoghurts produced with low- and high-protein content was evaluated on the 1st day of storage, and the results are presented in Table 1. Regarding the low-protein yoghurts, no differences were found between samples in ash, fat and protein content. However, yoghurts produced with sucrose had a significantly higher content of total solids and carbohydrates (14.8 and 10.2%, respectively), while stevia presented the lowest values (9.6 and 5.1%). These differences resulted from the distinct levels of sweetener addition (i.e., 6% for sucrose, 4.5% for agave and 0.03% for stevia), impacting total solids differently. Specifically, the addition of more sucrose resulted in a significant increase in total solids, which in turn led to a higher carbohydrate content. This is because carbohydrates are closely linked to total solid content, as shown in Equation (1). For the high-protein yoghurts, no differences were found in fat and protein content between samples. Yoghurts sweetened with sucrose and xylitol had a higher total solids content (20.9% and 20.4%, respectively), compared to honey (19.2%)- and stevia (14.8%)-sweetened yoghurts. Although the levels of addition of sucrose and honey were similar, yoghurts sweetened with honey had slightly lower levels of solids, most probably due the fact that honey has also a lower solids content than the other two sweeteners. Yoghurts produced with xylitol had a much lower ash content (0.59%) compared to those produced with sucrose, stevia and honey (≈0.90%). This finding is supported by Costa et al. [10], who also observed a lower ash content in yoghurts sweetened with xylitol (1.58%) compared to those sweetened with sucrose (2.29%). Regarding carbohydrates, sucrose-, xylitol- and honey-sweetened yoghurts presented no differences, with high carbohydrates content (≈13%), while stevia-sweetened yoghurts had a much lower value of 6.4%. These variations in carbohydrate content align with the concentrations of the sweeteners used, as mentioned before for the low-protein yoghurts. In both trials, the yoghurts containing sucrose had the highest total solids and carbohydrates content, while those containing stevia had the lowest ones.

Most studies on yoghurts have been conducted using normal levels of fat. In their study, Costa et al. [10] tested stevia A and B (0.6 g/L) and xylitol (120 g/L) as sweeteners in yoghurts containing 3.37–3.72% fat and 2.87–3.18% protein, comparing them to yoghurts sweetened with sucrose (120 g/L). They found that yoghurts sweetened with sucrose had higher levels of total solids (26%) and carbohydrates (18%), followed by xylitol, which presented similar results (24% for total solids and 16% for carbohydrates). The yoghurts sweetened with stevia had the lowest amount of total solids (15%) and carbohydrates (6%). Machado et al. [27] used honey as a sweetener in yoghurts, resulting in a fat, protein and total solids contents of 2.75%, 3.93% and 16.9%, respectively. The author’s results agree with those found in the present study for the high-protein yoghurts, despite the differences in fat and protein content. The sucrose- and xylitol-sweetened samples presented similar total solids and carbohydrate amounts, followed by honey and stevia (Table 1). Although García et al. [13] focused on using agave (6%) as a fat replacer in yoghurts (1% of fat), the products they obtained had a total solids content (10.5%) similar to that of the yoghurts produced in the present study (12.2%).

Generally, lactic acid bacteria ferment lactose into lactic acid [28], which can alter the pH and titratable acidity of yoghurt over time. As lactic acid accumulates in yoghurt, the pH typically decreases and the titratable acidity increases. The rate and extent of pH and titratable acidity changes in yoghurt can be affected by several variables, such as the starting pH of the milk, the type and concentration of starter culture used, the fermentation temperature and time and the presence of other additives such as sweeteners [28,29,30].

**Figure 1:**
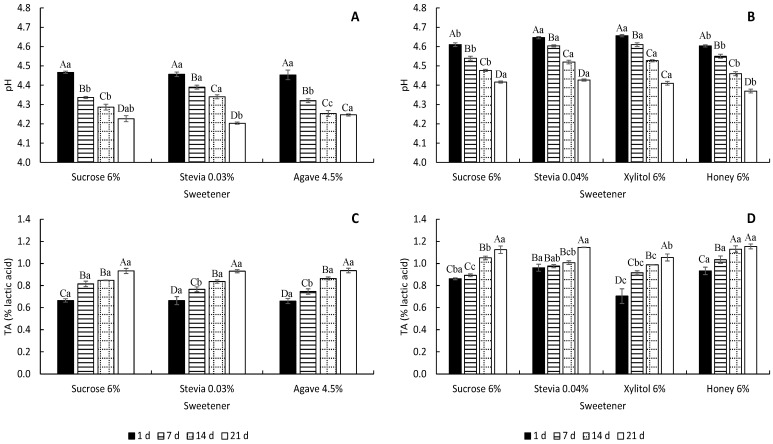
pH (**A**,**B**) and titratable acidity (**C**,**D**) (mean ± standard deviation) of the low-fat and low-protein yoghurts (**A**,**C**) and high-protein yoghurts (**B**,**D**) produced with different sweeteners. Different capital letters within each sweetener represent statistical differences between storage time for each parameter. Different small letters within each storage time represent statistical differences between sweeteners for each parameter (*p* < 0.05).

Figure 1 presents the pH and titratable acidity results for the low-fat, low-protein and high-protein yoghurts. The pH values (Figure 1A,B) decreased with storage time, accompanied by an increase in titratable acidity over time in all yoghurts (Figure 1C,D), which is an expected trend in fermented dairy products. The pH and titratable acidity of yoghurts changed during storage due to the availability of energy sources for the activity of starter microorganisms. This activity persists despite the low storage temperature of yoghurts. It is worth noting that the high-protein yoghurts had a higher pH despite having higher acidity values. This is likely due to the buffering effect of proteins, which are present in higher amounts in these samples. On the 21st day, the low-protein yoghurts with the natural sweeteners stevia and agave were not significantly different from those with sucrose, with a pH of 4.2 and a titratable acidity of 0.93% (Table S1). However, the pH of the high-protein yoghurts sweetened with honey was the lowest (4.4), and the titratable acidity of the yoghurts produced with xylitol was also statistically different from the other sweeteners (1.05% lactic acid) (Table S2). On the 21st day of storage, despite significant differences, the pH and titratable acidity of the low-protein yoghurts varied between 4.20 and 4.25 and 0.93 and 0.94% lactic acid, respectively. For the high-protein yoghurts, the pH varied between 4.37 and 4.43, and the titratable acidity varied from 1.05 to 1.15% lactic acid. It appears that the sweeteners did not have a considerable effect on the acidity of the yoghurts.

The results are similar to those found in other studies. Costa et al. [10] evaluated the pH and titratable acidity in yoghurts sweetened with sucrose, stevia and xylitol, on the 1st and 21st day of storage. Over storage, the pH decreased from 4.40 to 4.20 in stevia yoghurts, while in sucrose- and xylitol-sweetened yoghurts, it decreased from 4.30 to 4.22 and 4.14, respectively. However, the titratable acidity only increased in yoghurts sweetened with stevia (0.85 to 0.95%). In yoghurts sweetened with sucrose and xylitol, the titratable acidity decreased from 0.82 to 0.80 and 0.81 to 0.71% lactic acid, respectively. The results for the yoghurts sweetened with stevia on the 21st day are more similar to the findings in the present study for the low-fat, low-protein sweetened yoghurts, than for the high-protein yoghurts. Machado et al. [27] evaluated honey-sweetened yoghurts on the 1st and 21st day of storage. The study found that the pH decreased from 4.57 to 4.40 with storage time, while the titratable acidity increased from 1.09 to 1.11% lactic acid. These results are consistent with those presented in Figure 1B,D for the 21st day of storage, where the pH of honey-sweetened yoghurts was 4.37 and the titratable acidity was 1.15% lactic acid.

**Figure 2:**
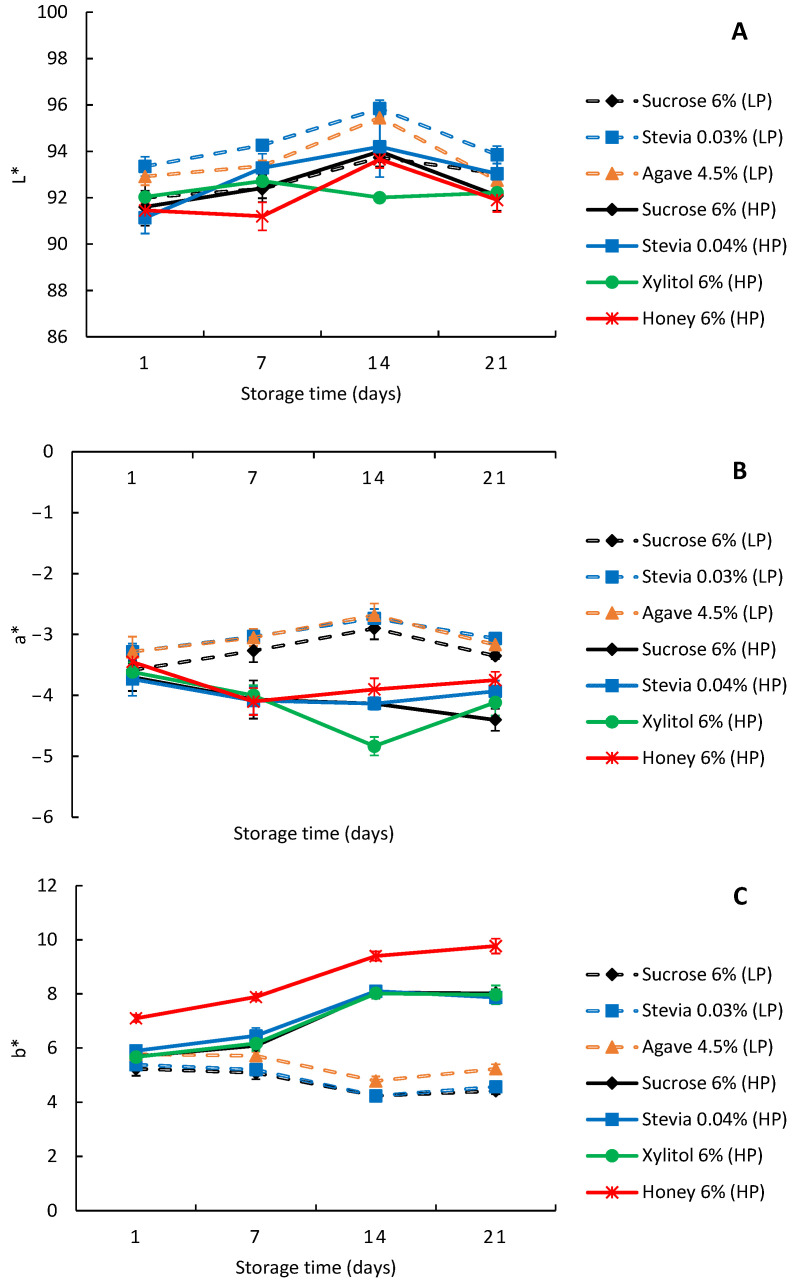
Color parameters L* (**A**), a* (**B**) and b* (**C**) (mean ± standard deviation) of the low-fat and low-protein yoghurts (dotted line) and high-protein yoghurts (full line) produced with different sweeteners. LP—low-protein; HP—high-protein.

The yoghurt color was determined using the L*a*b* coordinate system. The L* coordinate indicates brightness, ranging from 0 (black) to 100 (white). The a* coordinate represents the green (negative values) to red (positive values) axis, while the b* coordinate represents the blue (negative values) to yellow (positive values) axis. Neutral grey is represented by zero on these axes. All evaluated yoghurts (Figure 2) had high brightness values (L* greater than 90), approaching a white color (Figure 2A). The a* (Figure 2B) and b* (Figure 2C) axis were close to zero, tending towards more yellowish and whitish shades. Statistically significant differences (*p* < 0.001) were found in the color parameters of yoghurts produced with different sweeteners over storage time (refer to Tables S3 and S4). Although the values for each trial (low- and high-protein) are very similar between the natural sweeteners and the sucrose control, statistically significant differences were also found (*p* < 0.001). The low- and high-protein yoghurts presented similar values between 91.6 and 95.9 for the L* axis. On the 1st day, all yoghurts exhibit a similar color with an a* value of approximately −3.5 and a b* value around 5.7. However, as time passed, the a* value remained stable in low-protein yoghurts, while in high-protein yoghurts, it decreased to a range between −3.8 (honey) and −4.4 (sucrose) by the 21st day. In terms of b* coordinate, low-protein yoghurts decreased to a range between 4.4 (sucrose) and 5.2 (agave), whereas high-protein yoghurts show an increase in b* values ranging from 7.9 (stevia) to 9.8 (honey) by the same day. This indicates that high-protein yoghurts deviate more from white and tend towards a yellowish hue. As expected, this trend was especially noticeable in the high-protein yoghurts sweetened with honey.

The difference between the a* and b* axis in the low- and high-protein yoghurts may be related to the Maillard reaction, which involves amino acids and reducing sugars [31]. This reaction can lead to the development of brown pigments, contributing to the yellow color of the yoghurt. Yoghurts with higher protein content may become more intensely yellow over time due to their higher concentration of amino acids available to participate in the Maillard reaction.

Costa et al. [10] and Machado et al. [27] evaluated the color parameters of yoghurts sweetened with sucrose, stevia, xylitol and honey. The L* value was below 90 for all yoghurts. Additionally, for sucrose, stevia and xylitol, the a* value was greater than 2.82 and the b* value was higher than 8.34 [10]. These values suggest that these yoghurts have a light-yellow color with some reddish or orange and yellowish or greenish undertones. Regarding the yoghurts analyzed in this study, their hue is best described as a very light green with some greenish or bluish and yellowish or greenish undertones. This hue is comparable to the yoghurts sweetened with xylitol from the study by Machado et al. [27], which had a* (−2.75) and b* (6.62) values, similar to those shown in Figure 2.

Syneresis is the process by which whey or water is released from a gel-like substance, such as yoghurt [32]. Syneresis can cause an increase in hardness due to whey expulsion from the gel structure, resulting in a denser protein matrix. This can lead to a texture that may be perceived as more solid and less creamy. When whey is expelled, it may also remove flavoring substances, resulting in a loss of flavor in the yoghurt. Moreover, water loss can cause a decrease in volume, making the yoghurt less appealing to consumers [33,34]. To prevent or reduce syneresis, increasing total solids and protein content improve gel firmness and water retention [35].

**Figure 3:**
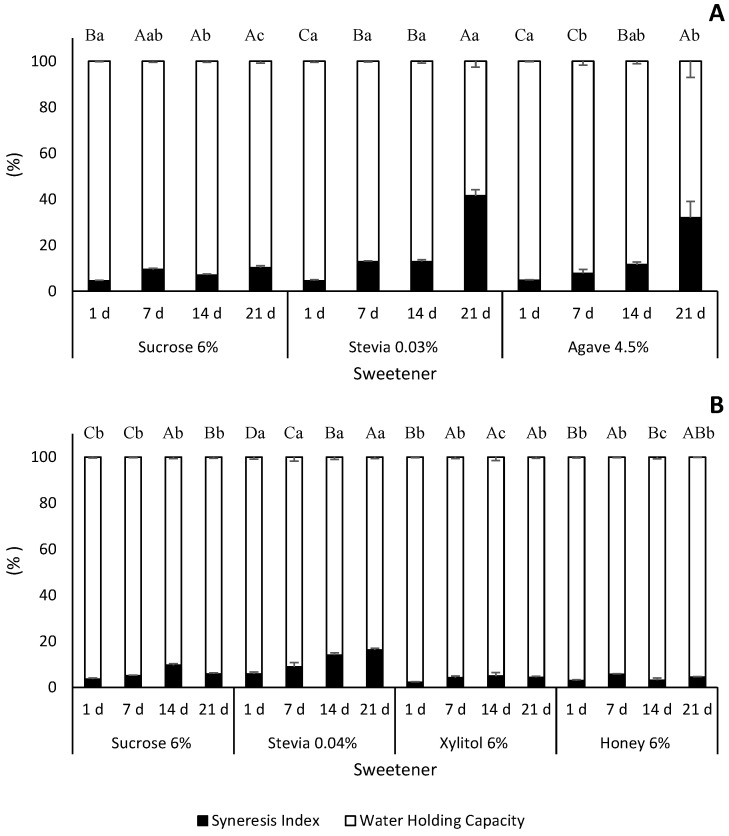
Syneresis index and water holding capacity (mean ± standard deviation) of the low-fat, low-protein yoghurts (**A**) and high-protein yoghurts (**B**) produced with different sweeteners. Different capital letters within each sweetener represent statistical differences between storage time. Different small letters within each storage time represent statistical differences between sweeteners (*p* < 0.05).

Throughout all storage time, high-protein yoghurts consistently demonstrated a lower syneresis index when compared to the low-protein yoghurts (Figure 3). On the 21st day of storage, low-protein yoghurts had a syneresis index of 10.2% for sucrose, 31.9% for agave and 41.5% for stevia. In contrast, high-protein yoghurts had a syneresis index of 5.7% for sucrose, 4.4% for xylitol and honey and 16.2% for stevia. In the high-protein yoghurts, xylitol and honey increased the water holding capacity to over 95%, compared to the sucrose yoghurts which had 90–96%. However, there were no statistically significant differences between the three types of yoghurts on the 1st, 7th and 21st days (Figure 3A). The increase in water holding capacity, and consequently the decrease in syneresis, may indicate greater product stability over storage [32]. This was also observed in the cases of xylitol and honey. Significant differences (*p* < 0.0001) were found for both low- and high-protein yoghurts regarding storage time and type of sweetener (Tables S5 and S6).

These findings are in accordance with the literature. The protein content has an impact on syneresis because it is crucial for the formation of yoghurt’s gel-like structure. The product is held together by a grid of intermolecular connections created by the milk proteins. During the manufacturing of yoghurt, the proteins denature and coagulate to form a three-dimensional network that traps the water and other components. However, if the protein concentration is too low or if the proteins are degraded during processing, the network may be weaker, resulting in a higher syneresis and lower water holding capacity [33,36,37,38]. Our study aligns with these established principles. We observed that low-protein yoghurts exhibited higher syneresis compared to high-protein yoghurts. This difference can be attributed to inadequate protein content, which prevents the formation of a robust network capable of retaining water. The present results demonstrate the importance of protein concentration in determining the quality and characteristics of yoghurt, particularly in relation to syneresis. Machado et al. [27] conducted a study on goat yoghurts sweetened with honey (5%, *v*/*v*) and evaluated their syneresis and water holding capacity. The results showed that there was no significant variation in these properties from the 1st to the 21st day of storage. However, the syneresis index presented in the study (around 50%) differs significantly from the values shown in Figure 3 (ranging from 2.8% on day 1 to 4.4% on day 21). Although the protein level in the formulations cannot fully explain these differences due to the similar protein content between sweetened goat yoghurts and low-protein yoghurts produced in the present study, the origin of the milk and the dimension of casein micelles of goat’s milk may also play an important role. Domagała [35] found that goat milk yoghurt is more susceptible to syneresis than cow’s and sheep’s milk yoghurts. This means that the microstructure of goat’s milk yoghurt is more delicate and less resistant to deformation compared to cow’s milk yoghurt.

**Figure 4:**
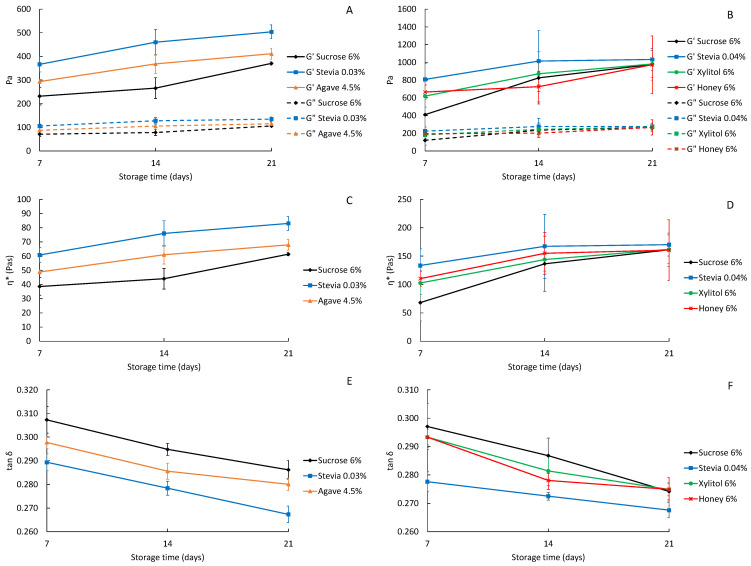
Elastic (G′) and viscous modulus (G″) (**A**,**B**), complex viscosity (η*) (**C**,**D**) and tan δ (**E**,**F**), at 1 Hz and 5 °C, of the low-fat and low-protein yoghurts (**A**,**C**,**E**) and high-protein yoghurts (**B**,**D**,**F**) produced with different sweeteners.

Rheological analysis was conducted on the 7th day after production, concurrently with the sensory analysis, and again on the 14th and 21st days of storage. The elastic modulus (G′), viscous modulus (G″), complex viscosity (η*) and the damping factor (tan δ) were evaluated at frequencies ranging from 0.05 to 1.00 Hz. Figure 4 presents the values of G′, G″, η* and tan δ at 1 Hz for comparison. The results presented in Figure 4A,B show that in all yoghurts, G′ was higher than G″, indicating that the yoghurts are weak viscoelastic gels and that their elastic characteristic is stronger than their viscous characteristic. Both G′ and G″ increased with time during storage, but this trend was more pronounced for G′. The yoghurts with higher protein content exhibited higher values of G′ and G″ compared to the lower protein content yoghurts. This is likely due to the protein’s significant role in forming the gel network in yoghurts, as previously stated. The present results for the elastic and viscous moduli in yoghurts with low and high-protein content agree with the results obtained for the syneresis index. The gel structure in high-protein yoghurts is more compact, which decreases syneresis and increases the G′ values. The complex viscosity (Figure 4C,D) follows the same behavior as the elastic modulus for both low- and high-protein yoghurts. This is because the elastic modulus is proportional to the product of the complex viscosity and the frequency (in rad/s). In both low- and high-protein yoghurts, the control group, with sucrose, presented the lowest values for G′, G″ and η*, while the stevia-sweetened yoghurts presented the highest values. The higher syneresis index observed in low-protein yoghurts sweetened with stevia and agave, and the consequent water expulsion from the protein matrix, originated the higher elastic moduli in such yoghurts. In the case of high-protein yoghurts sweetened with stevia, the same pattern is observed, although differences between sweeteners are less evident at the end of storage.

The damping factor is defined as the ratio of the viscous (G″) to elastic (G′) response (Figure 4E,F). The tan δ decreases with time because the G′ modulus has a more significant increase over time than the G″ modulus. The tan δ value helps to determine whether the yoghurt has a predominant viscous or elastic characteristic [39]. If the value of tan δ is close to 1, it indicates that the material is balanced between its viscous and elastic response. On the other hand, if the value of tan δ is close to zero, it suggests that the material is more elastic than viscous. The study suggests that yoghurts tend to become more elastic over time as the tan δ decreases with storage. Sucrose-sweetened yoghurts presented the highest values for tan δ, while stevia-sweetened yoghurts presented the lowest values as a result of their higher syneresis index. In the case of low-protein yoghurts the ones sweetened with agave presented intermediate results as a result of the loss of water and increased G′ values, while in high-protein yoghurts differences were not observed between sucrose, xylitol and honey after 14 days of storage.

Significant differences were found between storage times (*p* < 0.05) for both low- and high-protein yoghurts in all parameters (G′, G″, η* and tan δ) (Tables S7 and S8). Additionally, significant differences between sweeteners were found only in low-protein yoghurts (*p* < 0.05).

Table 2 contains the parameters for the power law equation. Higher levels of “*a*” (consistency index) represent the ability of the yoghurts to form a strong gel structure, while high values of “*b*” (slope of the curve) represent a high sensitivity to mechanical stress [39]. The “*a*” factor increased over storage time in the yoghurts. The high-protein yoghurts also exhibited a higher consistency index, which is consistent with previous findings that suggest a more compact gel structure in high-protein yoghurts. In both low- and high-protein yoghurts, those sweetened with sucrose had the lowest consistency, while those sweetened with stevia had a stronger gel structure. Again, the loss of water from the protein matrix can be considered the main factor affecting the consistency of yoghurt samples.

The sensitivity to mechanical stress “*b*” decreased over time during storage, except for high-protein yoghurts sweetened with stevia. The values of “*b*” for both low- and high-protein yoghurts are very similar, as are the values between the sweeteners.

**Figure 5:**
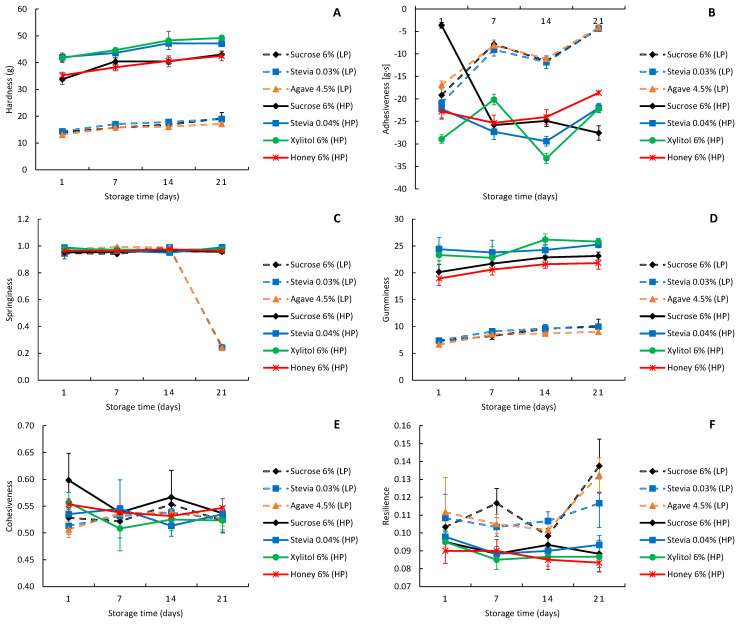
Texture parameters: hardness (**A**), adhesiveness (**B**), springiness (**C**), gumminess (**D**), cohesiveness (**E**) and resilience (**F**) (mean ± standard deviation) of the low-fat and low-protein yoghurts (dotted line) and high-protein yoghurts (full line) produced with different sweeteners. LP—low-protein; HP—high-protein.

For texture analysis, hardness, adhesiveness, springiness, gumminess, cohesiveness and resilience of the yoghurts were evaluated throughout the 21 days of storage (Figure 5). The graphical representation of the differences (*p* < 0.05) between low- and high-protein yoghurts in terms of their hardness (Figure 5A), adhesiveness (Figure 5B) and gumminess (Figure 5D) evidences the differences. Additionally, an upward trend in the values of these parameters was observed among the sweetened yoghurt types and over time (Tables S9 and S10).

As previously stated, syneresis can directly impact the texture of yoghurts, as higher syneresis can lead to increased hardness. This observation justifies the increase in hardness of both types of yoghurts over time. However, the difference in protein content between low- and high-protein yoghurts is clearly related to the higher density of the protein matrix in the latter. The higher hardness values of these samples are in line with previous findings, where high-protein yoghurts exhibited higher G′, indicating a stronger gel structure.

The yoghurts with low-protein content showed higher adhesiveness (between −4 and −21 g·s) than the high-protein yoghurts (<−20 g·s, with the exception of sucrose-sweetened high-protein yoghurt on the 1st day of storage) (Figure 5B). Another parameter that showed significant differences between low- and high-protein yoghurts was gumminess (Figure 5D). In this case, high-protein yoghurts had higher gumminess (>20 g) than the low-protein yoghurts (<10 g).

Regarding springiness (Figure 5C), cohesiveness (Figure 5E) and resilience (Figure 5F), in most cases, significant differences were found between yoghurts of the same type and as a result of storage time (*p* < 0.05, Tables S9 and S10). However, the overall results for the low-protein and high-protein yoghurts are very similar between all samples. The results shows that springiness ranged from 0.94 to 0.99, except for low-protein yoghurts on the 21st day of storage, where it decreased abruptly to 0.25. Cohesiveness and resilience remained stable throughout the storage time, with cohesiveness ranging from 0.51 to 0.60 and resilience from 0.08 to 0.14.

Costa et al. [10] compared sweetened yoghurts with stevia, xylitol and sucrose. The firmness of the sucrose yoghurts was the lowest of the three, starting at 69.9 g on the 1st day and slightly decreasing to 67.9 g on the 21st day. Xylitol scored higher, with 70.1 g on day 1 and increasing to 72.9 g on day 21. Stevia yoghurts, which were sweetened with stevia A and B, respectively, started at 95.4 g and 85.5 g and decreased the most, reaching the values of 65.5 g and 60.6 g on the 21st day of storage. Although Costa et al. [10] reported higher results compared to our study, it is important to note that the high-protein yoghurts showed similar hardness to their findings. This highlights a relative consistency in the textural attributes despite different experimental conditions. The authors concluded that xylitol-sweetened yoghurts showed similar results to sucrose-sweetened yoghurts on the 1st day of storage. Xylitol yoghurts were more effective in maintaining textural characteristics, while stevia yoghurts presented significantly higher values, resulting in decreased consumer acceptance. In the present study, that was not verified, since the low-protein yoghurts showed small differences between sweeteners, while the high-protein yoghurts showed that sucrose and honey yoghurts were very similar, and stevia and xylitol presented higher hardness.

The observed differences in rheological and textural properties between low-protein and high-protein yoghurts sweetened with various natural alternatives to sucrose highlight the significant impact of protein content on the final product. Higher protein levels are consistently associated with a firmer yoghurt structure, as proteins play a crucial role in forming and stabilizing the gel matrix. The present study provides evidence that high-protein level in the formulation of yoghurts contributes to enhanced firmness. This finding aligns with established knowledge in dairy science, underlining the fundamental role of protein in shaping the structural attributes of yoghurts. Therefore, the differences in texture observed among the yoghurts can be attributed to variations in protein content, highlighting its crucial role in determining the final texture of the product. Moreover, the increase in syneresis observed in stevia-sweetened yoghurts also played a major role on the rheological and textural parameters of yoghurts. Ultimately, the lower water retention capacity of such yoghurts is related to their lower level of solids, and this fact has to be considered when preparing the formulation.

**Figure 6:**
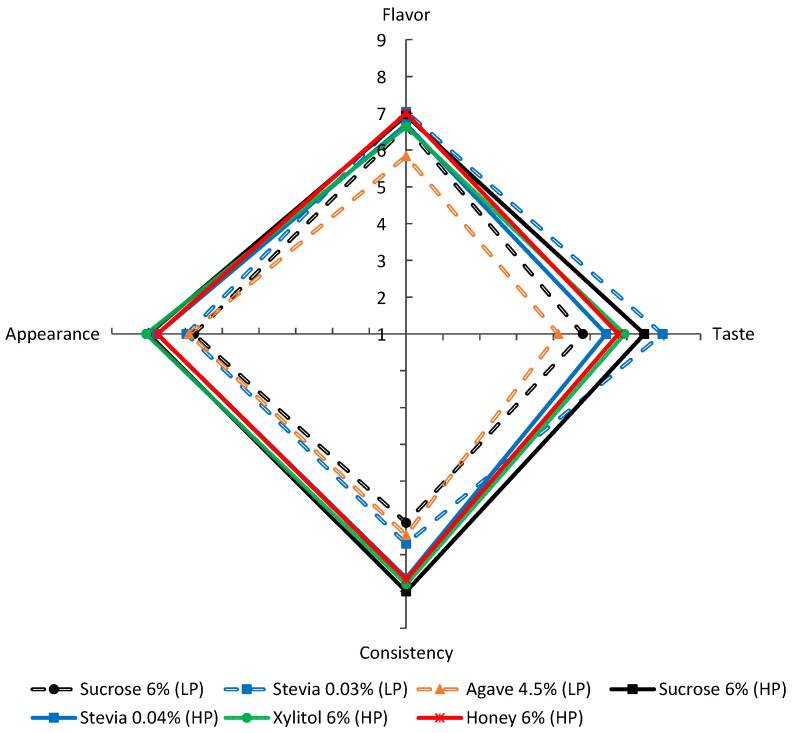
Sensorial analysis (mean values) of the low-fat and low-protein yoghurts (dotted line) and high-protein yoghurts (full line) produced with different sweeteners. LP—low-protein; HP—high-protein.

On the 7th day of storage, 33 untrained panelists evaluated the flavor, taste, consistency and appearance of all sweetened yoghurts (Figure 6). Statistically significant differences were found in all parameters (Table S11). The high-protein yoghurts scored higher than the low-protein yoghurts, particularly regarding appearance and consistency (score > 7.5). The yoghurts with low protein produced with stevia scored highest regarding flavor (7.0) and taste (8.0). High-protein yoghurts made with xylitol scored highest on consistency (7.8) and appearance (8.1). Chadha et al. [2] found that consumers did not detect any negative sensory characteristics in yoghurts produced with xylitol, which had acceptance results like those found for sucrose. In contrast, yoghurts produced with stevia were considered astringent. This may also be the case in the present study, as the high-protein yoghurts sweetened with stevia scored the lowest (6.4) in taste and had a higher concentration of stevia than the low-protein yoghurts. According to the authors, xylitol- and stevia-sweetened yoghurts were found to reduce consumers’ hunger compared to sucrose [2].

Several authors compared the acceptance of yoghurts produced with various natural sweeteners using a nine-point scale. In their study, Costa et al. [10] compared the flavor, appearance, consistency and aroma of sucrose-sweetened yoghurts and stevia- and xylitol-sweetened yoghurts. Sucrose yoghurts received the highest scores in all parameters, followed by xylitol and stevia yoghurts. The results showed a significant difference in flavor perception among the three types of yoghurt. Specially, the stevia-sweetened yoghurt received a score of 5.0, while the xylitol yoghurt received a score of 7.7 and the sucrose received a score of 8.1. Tondare and Hembade [40] conducted a similar study comparing sucrose and stevia and also found that sucrose scored higher than stevia in flavor (7.8 to 6.5), taste (8.2 to 7.6), appearance (7.7 to 6.3) and consistency (7.5 to 6.8). Machado et al. [27] evaluated the acceptance of honey-sweetened yoghurts and found that they scored 5.3 in flavor, 6.9 in appearance and 6.5 in consistency.

De Carvalho et al. [11] conducted a survey on the acceptance of yoghurts produced with sucrose, xylitol and stevia. The authors concluded that participants preferred yoghurts sweetened with sucrose for their “sweet taste” and “creamy texture” more often than those sweetened with xylitol and stevia. These results are close to those found in this study for high-protein yoghurts, considering taste and consistency evaluation, since sucrose yoghurts scored the highest, followed by xylitol, honey and stevia. In contrast, low-protein yoghurts in the present study were preferred when sweetened with stevia over sucrose and agave.

In a study conducted by other authors [41], products sweetened with sucrose, stevia and honey were evaluated for their perceived healthfulness based on their labels. The products labeled as “stevia” were rated as more healthy but less tasty. Among sweeteners, honey is considered the second healthiest option and is perceived to have the best taste by consumers. On the other hand, sucrose received the lowest score for healthfulness and was ranked second in terms of taste. Considering taste, our findings indicate that in the low-protein yoghurts, stevia received the highest score (8.0), while in the high-protein yoghurts, sucrose received the highest score (7.5) and stevia received the lowest score (6.4). Two main factors may have contributed to these results. The yoghurt samples with low- and high-protein content were evaluated separately by the panelists. Although both trials used sucrose and stevia, the remaining sweeteners differed, resulting in a change in the panelists’ relative perception of the characteristics of the yoghurt. Secondly, the intensity of the stevia in yoghurt appears to vary with the levels of protein and total solids content. At low levels of protein and total solids, stevia performs better than sucrose. However, at higher levels, even with a higher concentration of stevia (0.04%), the taste of the samples was negatively affected. Further research is needed to determine the optimal concentration and type of sweetener to achieve the same level of satisfaction as sucrose.

## 4. Conclusions

This study assessed the impact of various natural sweeteners on the physicochemical and sensory characteristics of low-fat yoghurts, while considering changes in protein content. The results revealed notable differences in syneresis, color, rheological properties and sensory attributes among yoghurts with different protein content. Low-protein yoghurts exhibited higher syneresis, while high-protein variants demonstrated a more compact gel structure, which influenced their texture and sensory acceptance. Color variations in the a* and b* parameters were likely associated with the Maillard reaction, which potentially intensified yellow in high-protein yoghurts due to increased amino acid content. When considering natural sweeteners, it is important to note that the choice of sweetener could have an impact on the overall characteristics of yoghurt. High-protein yoghurts sweetened with xylitol or honey scored higher in appearance and consistency, while low-protein yoghurts sweetened with stevia stood out in terms of flavor and taste. To improve consumer preference for low-fat yoghurt, it is recommended to increase the total solids and protein contents in the formulations, regardless of the type of sweetener used. This will help to overcome the lack of fat.

The yoghurt sweetened with xylitol appears to be the best formulation due to its high nutritional value, including a high-protein content, and positive physicochemical characteristics. It was also the most appreciated sample.

This study opens the possibility for future research into optimal combinations of sweeteners and protein content and their effects on yoghurt attributes.

## Figures and Tables

**Table 1:** Chemical composition (mean ± standard deviation) of the low-fat and low-protein yoghurts and high-protein yoghurts produced with different sweeteners. Different small letters within the same column represent statistical differences between sweeteners of the low-protein yoghurts. Different capital letters within the same column represent statistical differences between sweeteners of the high-protein yoghurts (*p* < 0.05).

Protein Content	Sweetener	Total Solids (%)	Ash (%, DM)	Fat (%, DM)	Protein (%, DM)	Carbohydrates (%, DM)
**Low protein**	Sucrose 6%	14.8 ± 0.0 ^a^	0.64 ± 0.09 ^a^	0.90 ± 0.00 ^a^	2.97 ± 0.13 ^a^	10.2 ± 0.2 ^a^
	Stevia 0.03%	9.6 ± 0.1 ^c^	0.60 ± 0.09 ^a^	0.83 ± 0.06 ^a^	3.12 ± 0.06 ^a^	5.1 ± 0.0 ^c^
	Agave 4.5%	12.2 ± 0.0 ^b^	0.65 ± 0.07 ^a^	0.90 ± 0.10 ^a^	3.06 ± 0.16 ^a^	7.6 ± 0.0 ^b^
	***p-value***	*<0.0001*	0.7015	0.4219	0.5400	*<0.0001*
**High protein**	Sucrose 6%	20.9 ± 0.2 ^A^	0.93 ± 0.01 ^A^	1.13 ± 0.06 ^A^	4.96 ± 0.46 ^A^	13.9 ± 0.1 ^A^
	Stevia 0.04%	14.8 ± 0.5 ^C^	0.94 ± 0.04 ^A^	1.17 ± 0.06 ^A^	6.45 ± 1.35 ^A^	6.4 ± 0.7 ^B^
	Xylitol 6%	20.4 ± 0.0 ^A^	0.59 ± 0.02 ^B^	1.23 ± 0.06 ^A^	4.62 ± 0.12 ^A^	13.9 ± 0.2 ^A^
	Honey 6%	19.2 ± 0.2 ^B^	0.86 ± 0.13 ^A^	1.20 ± 0.10 ^A^	4.36 ± 0.74 ^A^	12.8 ± 0.7 ^A^
	***p-value***	*<0.0001*	*0.0007*	0.3999	0.1837	*0.0003*

**Table 2:** Power law equation parameters: “*a*” (consistency index) and “*b*” (slope of the curve) (mean ± standard deviation) of the low-fat, low-protein yoghurts and high-protein yoghurts produced with different sweeteners. Different capital letters within the same column represent statistical differences between storage time for each parameter in the same sweetener. Different small letters within the same row represent statistical differences between sweeteners for each parameter in the same storage time (*p* < 0.05).

**Low-Fat, Low-Protein Yoghurts**							
**Parameter**	**Storage Time (Days)**	**Sucrose 6%**	**Stevia 0.03%**	**Agave 4.5%**	*-*	***p-Value***	
*a*	7	236.0 ± 37.9 ^Bb^	373.5 ± 33.3 ^Ba^	298.5 ± 8.3 ^Bb^	-	Sweetener (A)	*<0.0001*
	14	269.5 ± 45.8 ^Bc^	467.3 ± 55.6 ^Aa^	374.6 ± 40.9 ^Ab^	-	Time (B)	*<0.0001*
	21	377.1 ± 8.5 ^Ab^	512.5 ± 30.2 ^Aa^	417.2 ± 22.7 ^Ab^	-	A × B	0.4285
*b*	7	0.212 ± 0.002 ^Aa^	0.203 ± 0.003 ^Aa^	0.204 ± 0.005 ^Aa^	-	Sweetener (A)	*0.0205*
	14	0.194 ± 0.013 ^Ba^	0.189 ± 0.003 ^Ba^	0.197 ± 0.006 ^ABa^	-	Time (B)	*<0.0001*
	21	0.197 ± 0.006 ^Ba^	0.186 ± 0.006 ^Ba^	0.188 ± 0.001 ^Ba^	-	A × B	0.4086
**Low-Fat, High-Protein Yoghurts**							
**Parameter**	**Storage Time (Days)**	**Sucrose 6%**	**Stevia 0.04%**	**Xylitol 6%**	**Honey 6%**	***p-Value***	
*a*	7	415.9 ± 199.0 ^Ba^	821.2 ± 184.1 ^Aa^	631.6 ± 127.8 ^Aa^	673.6 ± 76.1 ^Aa^	Sweetener (A)	0.4223
	14	842.8 ± 303.5 ^ABa^	1030.6 ± 349.6 ^Aa^	888.5 ± 125.4 ^Aa^	773.5 ± 183.7 ^Aa^	Time (B)	0.3803
	21	994.8 ± 180.6 ^Aa^	1050.7 ± 135.5 ^Aa^	1001.7 ± 154.4 ^Aa^	988.2 ± 329.6 ^Aa^	A × B	0.5527
*b*	7	0.210 ± 0.003 ^Aa^	0.199 ± 0.004 ^Ab^	0.214 ± 0.002 ^Aa^	0.218 ± 0.004 ^Aa^	Sweetener (A)	*0.0074*
	14	0.211 ± 0.003 ^Aa^	0.196 ± 0.004 ^Ab^	0.201 ± 0.001 ^Bab^	0.201 ± 0.005 ^Bab^	Time (B)	*<0.0001*
	21	0.199 ± 0.007 ^Ba^	0.201 ± 0.010 ^Aa^	0.197 ± 0.003 ^Ba^	0.197 ± 0.004 ^Ba^	A × B	*0.0035*

## Data Availability

The original contributions presented in the study are included in the article/Supplementary Material, further inquiries can be directed to the corresponding author.

## Supplementary Materials

The following supporting information can be downloaded at: https://www.mdpi.com/article/10.3390/foods13020250/s1. Table S1: pH and titratable acidity (TA, % lactic acid) (mean ± standard deviation) of the low-fat, low-protein yoghurts produced with different sweeteners. Different capital letters within the same column represent statistical differences between storage time for each parameter in the same sweetener. Different small letters within the same row represent statistical differences between sweeteners for each parameter in the same storage time (*p* < 0.05). Table S2: pH and titratable acidity (TA, % lactic acid) (mean ± standard deviation) of the low-fat, high-protein yoghurts produced with different sweeteners. Different capital letters within the same column represent statistical differences between storage time for each parameter in the same sweetener. Different small letters within the same row represent statistical differences between sweeteners for each parameter in the same storage time (*p* < 0.05). Table S3: Color parameters (L*, a*, b*) (mean ± standard deviation) of the low-fat, low-protein yoghurts produced with different sweeteners. Different capital letters within the same column represent statistical differences between storage time for each parameter in the same sweetener. Different small letters within the same row represent statistical differences between sweeteners for each parameter in the same storage time (*p* < 0.05). Table S4: Color parameters (L*, a*, b*) (mean ± standard deviation) of the low-fat, high-protein yoghurts produced with different sweeteners. Different capital letters within the same column represent statistical differences between storage time for each parameter in the same sweetener. Different small letters within the same row represent statistical differences between sweeteners for each parameter in the same storage time (*p* < 0.05). Table S5: Syneresis index and water holding capacity (mean ± standard deviation) of the low-fat, low-protein yoghurts produced with different sweeteners. Different capital letters within the same column represent statistical differences between storage time for each parameter in the same sweetener. Different small letters within the same row represent statistical differences between sweeteners for each parameter in the same storage time (*p* < 0.05). Table S6: Syneresis index and water holding capacity (mean ± standard deviation) of the low-fat, high-protein yoghurts produced with different sweeteners. Different capital letters within the same column represent statistical differences between storage time for each parameter in the same sweetener. Different small letters within the same row represent statistical differences between sweeteners for each parameter in the same storage time (*p* < 0.05). Table S7: Rheological parameters (mean ± standard deviation) of the low-fat, low-protein yoghurts produced with different sweeteners. Different capital letters within the same column represent statistical differences between storage time for each parameter in the same sweetener. Different small letters within the same row represent statistical differences between sweeteners for each parameter in the same storage time (*p* < 0.05). Table S8: Rheological parameters (mean ± standard deviation) of the low-fat, high-protein yoghurts produced with different sweeteners. Different capital letters within the same column represent statistical differences between storage time for each parameter in the same sweetener. Different small letters within the same row represent statistical differences between sweeteners for each parameter in the same storage time (*p* < 0.05). Table S9: Texture parameters (mean ± standard deviation) of the low-fat, low-protein yoghurts produced with different sweeteners. Different capital letters within the same column represent statistical differences between storage time for each parameter in the same sweetener. Different small letters within the same row represent statistical differences between sweeteners for each parameter in the same storage time (*p* < 0.05). Table S10: Texture parameters (mean ± standard deviation) of the low-fat, high-protein yoghurts produced with different sweeteners. Different capital letters within the same column represent statistical differences between storage time for each parameter in the same sweetener. Different small letters within the same row represent statistical differences between sweeteners for each parameter in the same storage time (*p* < 0.05). Table S11: Sensorial analysis results (mean ± standard deviation) regarding flavor, taste, consistency and appearance of the yoghurts produced with low-protein (LP) and high-protein (HP) and different sweeteners. Different small letters within the same row represent statistical differences between sweetened yoghurts for each parameter (*p* < 0.05).
