# Supplementary material for: Low Fat Yoghurts Produced with Different Protein Levels and Alternative Natural Sweeteners
Source: Foods. 2024 Jan 12;13(2):250. doi: 10.3390/foods13020250 (PMC10814987; doi:10.3390/foods13020250)
Supplement: Supplementary file 1 [file foods-13-00250-s001.zip › foods-2786109-supplementary.pdf]

Supplementary Material

Low Fat Yoghurts Produced with Different Protein Levels and Alternative Natural Sweeteners

Lara Campos [1], Paulina Tuma [2], Tânia Silva [3], David Gomes [1,3], Carlos Dias Pereira [1,3] and Marta H. F. Henriques [1,3*]

[1] Research Centre for Natural Resources Environment and Society (CERNAS), Coimbra Agriculture School, Bencanta, 3045-601 Coimbra, Portugal; lara.campos@esac.pt (LC); david@esac.pt (DG); cpereira@esac.pt (CDP);

[2] West Pomeranian University of Technology, Department of Food Technology and Human Nutrition, Szczecin, al. Piastów 42, 71-065 Szczecin, Poland; paulina272@gmail.com (PT);

[3] Polytechnic Institute of Coimbra, Coimbra Agriculture School, Bencanta, 3045-601 Coimbra, Portugal; tania.silva@esac.pt (TS)

[*] Correspondence: mhenriques@esac.pt

Table S1. pH and titratable acidity (TA, % lactic acid) (mean ± standard deviation) of the low-fat, low-protein yoghurts produced with different sweeteners. Different capital letters within the same column represent statistical differences between storage time for each parameter in the same sweetener. Different small letters within the same row represent statistical differences between sweeteners for each parameter in the same storage time ($p < 0.05$).

Parameter	Storage time (days)	Sucrose 6%	Stevia 0.03%	Agave 4.5%	*P-value*	
pH	1	4.47 ± 0.01^{Aa}	4.46 ± 0.01^{Aa}	4.45 ± 0.03^{Aa}		
	7	4.34 ± 0.01^{Bb}	4.39 ± 0.01^{Ba}	4.32 ± 0.01^{Bb}	Sweetener (A)<br>Time (B)<br>A x B	*<0.0001*<br>*<0.0001*<br>*<0.0001*
	14	4.29 ± 0.02^{Cb}	4.34 ± 0.01^{Ca}	4.25 ± 0.02^{Cc}		
	21	4.23 ± 0.02^{Dab}	4.20 ± 0.01^{Db}	4.25 ± 0.01^{Ca}		
TA (% lactic acid)	1	0.67 ± 0.02^{Ca}	0.66 ± 0.04^{Da}	0.66 ± 0.02^{Da}		
	7	0.82 ± 0.02^{Ba}	0.77 ± 0.02^{Cb}	0.75 ± 0.03^{Cb}	Sweetener (A)<br>Time (B)<br>A x B	0.1564<br>*<0.0001*<br>*0.0423*
	14	0.85 ± 0.00^{Ba}	0.84 ± 0.02^{Ba}	0.86 ± 0.01^{Ba}		
	21	0.93 ± 0.02^{Aa}	0.93 ± 0.01^{Aa}	0.94 ± 0.02^{Aa}		

Table S2. pH and titratable acidity (TA, % lactic acid) (mean ± standard deviation) of the low-fat, high-protein yoghurts produced with different sweeteners. Different capital letters within the same column represent statistical differences between storage time for each parameter in the same sweetener. Different small letters within the same row represent statistical differences between sweeteners for each parameter in the same storage time ($p < 0.05$).

Parameter	Storage time (days)	Sucrose 6%	Stevia 0.04%	Xylitol 6%	Honey 6%	*P-value*	
pH	1	4.61 ± 0.01^{Ab}	4.65 ± 0.01^{Aa}	4.66 ± 0.01^{Aa}	4.60 ± 0.01^{Ab}		
	7	4.54 ± 0.01^{Bb}	4.60 ± 0.01^{Ba}	4.61 ± 0.01^{Ba}	4.55 ± 0.01^{Bb}	Sweetener (A)<br>Time (B)<br>A x B	*<0.0001*<br>*<0.0001*<br>*<0.0001*
	14	4.48 ± 0.01^{Cb}	4.52 ± 0.01^{Ca}	4.53 ± 0.01^{Ca}	4.46 ± 0.01^{Cb}		
	21	4.42 ± 0.01^{Da}	4.43 ± 0.01^{Da}	4.41 ± 0.01^{Ca}	4.37 ± 0.01^{Db}		
TA (% lactic acid)	1	0.86 ± 0.01^{Cba}	0.96 ± 0.03^{Ba}	0.70 ± 0.07^{Dc}	0.93 ± 0.03^{Ca}		
	7	0.89 ± 0.01^{Cc}	0.98 ± 0.01^{Bab}	0.92 ± 0.02^{Cbc}	1.03 ± 0.03^{Ba}	Sweetener (A)<br>Time (B)<br>A x B	*<0.0001*<br>*<0.0001*<br>*<0.0001*
	14	1.05 ± 0.02^{Bb}	1.01 ± 0.02^{Bbc}	0.99 ± 0.00^{Bc}	1.13 ± 0.03^{Aa}		
	21	1.12 ± 0.03^{Aa}	1.15 ± 0.00^{Aa}	1.05 ± 0.03^{Ab}	1.15 ± 0.02^{Aa}		

Table S3. Color parameters (L*, a*, b*) (mean ± standard deviation) of the low-fat, low-protein yoghurts produced with different sweeteners. Different capital letters within the same column represent statistical differences between storage time for each parameter in the same sweetener. Different small letters within the same row represent statistical differences between sweeteners for each parameter in the same storage time ($p < 0.05$).

Parameter	Storage time (days)	Sucrose 6%	Stevia 0.03%	Agave 4.5%	*P-value*	
L*	1	92.0 ± 1.2^{Cb}	93.4 ± 0.4^{Ca}	92.9 ± 0.4^{Ba}	Sweetener (A)	*<0.0001*
	7	92.4 ± 0.6^{BCc}	94.3 ± 0.2^{Ba}	93.4 ± 0.2^{Bb}	Time (B)	*<0.0001*
	14	93.7 ± 0.4^{Ab}	95.9 ± 0.4^{Aa}	95.5 ± 0.3^{Aa}	A x B	*0.0007*
	21	93.1 ± 0.6^{ABb}	93.9 ± 0.4^{BCa}	92.8 ± 0.3^{Bb}		
a*	1	-3.6 ± 0.1^{Cb}	-3.3 ± 0.1^{Ca}	-3.3 ± 0.2^{Ba}	Sweetener (A)	*<0.0001*
	7	-3.3 ± 0.2^{Bb}	-3.0 ± 0.1^{Ba}	-3.1 ± 0.1^{Bab}	Time (B)	*<0.0001*
	14	-2.9 ± 0.2^{Aa}	-2.7 ± 0.2^{Aa}	-2.7 ± 0.2^{Aa}	A x B	0.8756
	21	-3.4 ± 0.1^{BCb}	-3.1 ± 0.1^{BCa}	-3.2 ± 0.2^{Bab}		
b*	1	5.2 ± 0.3^{Ab}	5.4 ± 0.2^{Ab}	5.8 ± 0.2^{Aa}	Sweetener (A)	*<0.0001*
	7	5.1 ± 0.2^{Ab}	5.2 ± 0.2^{Ab}	5.7 ± 0.1^{Aa}	Time (B)	*<0.0001*
	14	4.2 ± 0.1^{Bb}	4.2 ± 0.2^{Cb}	4.8 ± 0.2^{Ca}	A x B	0.3681
	21	4.4 ± 0.1^{Bb}	4.6 ± 0.1^{Bb}	5.2 ± 0.2^{Ba}		

Table S4. Color parameters (L*, a*, b*) (mean ± standard deviation) of the low-fat, high-protein yoghurts produced with different sweeteners. Different capital letters within the same column represent statistical differences between storage time for each parameter in the same sweetener. Different small letters within the same row represent statistical differences between sweeteners for each parameter in the same storage time ($p < 0.05$).

Parameter	Storage time (days)	Sucrose 6%	Stevia 0.04%	Xylitol 6%	Honey 6%	*P-value*	
L*	1	91.6 ± 0.7^{Ba}	91.1 ± 1.0^{Ca}	92.0 ± 0.7^{Aa}	91.5 ± 0.3^{Ba}	Sweetener (A)	*<0.0001*
	7	92.4 ± 0.4^{Ba}	93.3 ± 0.3^{ABa}	92.7 ± 0.6^{Ab}	91.2 ± 0.6^{Bb}	Time (B)	*<0.0001*
	14	94.0 ± 0.3^{Aa}	94.2 ± 0.5^{Aa}	92.0 ± 1.3^{Ab}	93.7 ± 0.4^{Aa}	A x B	*<0.0001*
	21	92.1 ± 0.6^{Bab}	93.0 ± 0.4^{Ba}	92.2 ± 0.4^{Aab}	91.9 ± 0.5^{Bb}		
a*	1	-3.7 ± 0.2^{Aa}	-3.7 ± 0.3^{Aa}	-3.6 ± 0.2^{Aa}	-3.5 ± 0.1^{Aa}	Sweetener (A)	*<0.0001*
	7	-4.1 ± 0.3^{Ba}	-4.1 ± 0.2^{Ba}	-4.0 ± 0.2^{Ba}	-4.1 ± 0.2^{Ca}	Time (B)	*<0.0001*
	14	-4.1 ± 0.1^{BCa}	-4.1 ± 0.1^{Ba}	-4.8 ± 0.2^{Cb}	-3.9 ± 0.2^{BCa}	A x B	*<0.0001*
	21	-4.4 ± 0.2^{Cc}	-3.9 ± 0.2^{ABab}	-4.1 ± 0.2^{Bbc}	-3.8 ± 0.1^{Ba}		
b*	1	5.7 ± 0.2^{Cb}	5.9 ± 0.2^{Cb}	5.7 ± 0.2^{Cb}	$7.1 \pm 0.1D^{a}$	Sweetener (A)	*<0.0001*
	7	6.1 ± 0.2^{Bc}	6.5 ± 0.3^{Bb}	6.2 ± 0.3^{Bbc}	7.9 ± 0.1^{Ca}	Time (B)	*<0.0001*
	14	8.1 ± 0.2^{Ab}	8.1 ± 0.2^{Ab}	8.0 ± 0.1^{Ab}	9.4 ± 0.2^{Ba}	A x B	*0.0037*
	21	8.0 ± 0.1^{Ab}	7.9 ± 0.2^{Ab}	8.0 ± 0.3^{Ab}	9.8 ± 0.3^{Aa}		

Table S5. Syneresis index and water holding capacity (mean ± standard deviation) of the low-fat, low-protein yoghurts produced with different sweeteners. Different capital letters within the same column represent statistical differences between storage time for each parameter in the same sweetener. Different small letters within the same row represent statistical differences between sweeteners for each parameter in the same storage time ($p < 0.05$).

Parameter	Storage time (days)	Sucrose 6%	Stevia 0.03%	Agave 4.5%	*P-value*	
Syneresis index (%)	1	4.6 ± 0.2^{Ba}	4.6 ± 0.5^{Ca}	4.8 ± 0.2^{Ca}		
	7	9.4 ± 0.5^{Aab}	12.9 ± 0.3^{Ba}	7.8 ± 1.7^{BCb}	Sweetener (A)	*<0.0001*
	14	7.1 ± 0.5^{Ab}	12.9 ± 0.9^{Ba}	11.6 ± 1.1^{Bab}	Time (B)	*<0.0001*
	21	10.2 ± 0.8^{Ac}	41.5 ± 2.6^{Aa}	31.9 ± 7.1^{Ab}	A x B	*<0.0001*
Water Holding Capacity (%)	1	95.4 ± 0.2^{Ba}	95.4 ± 0.5^{Ca}	95.2 ± 0.2^{Ca}		
	7	90.6 ± 0.5^{Aab}	87.1 ± 0.3^{Ba}	92.2 ± 1.7^{BCb}	Sweetener (A)	*<0.0001*
	14	92.9 ± 0.5^{Ab}	87.1 ± 0.9^{Ba}	88.4 ± 1.1^{Bab}	Time (B)	*<0.0001*
	21	89.8 ± 0.8^{Ac}	58.5 ± 2.6^{Aa}	68.1 ± 7.1^{Ab}	A x B	*<0.0001*

Table S6. Syneresis index and water holding capacity (mean ± standard deviation) of the low-fat, high-protein yoghurts produced with different sweeteners. Different capital letters within the same column represent statistical differences between storage time for each parameter in the same sweetener. Different small letters within the same row represent statistical differences between sweeteners for each parameter in the same storage time ($p < 0.05$).

Parameter	Storage time (days)	Sucrose 6%	Stevia 0.04%	Xylitol 6%	Honey 6%	*P-value*	
Syneresis index (%)	1	3.6 ± 0.4^{Cb}	5.7 ± 0.9^{Da}	2.1 ± 0.3^{Bb}	2.8 ± 0.4^{Bb}		
	7	5.0 ± 0.3^{BCb}	8.9 ± 1.8^{Ca}	4.1 ± 0.7^{Ab}	5.5 ± 0.3^{Ab}	Sweetener (A)	*<0.0001*
	14	9.6 ± 0.7^{Ab}	14.0 ± 1.0^{Ba}	4.9 ± 1.5^{Ac}	3.1 ± 0.8^{Bc}	Time (B)	*<0.0001*
	21	5.7 ± 0.5^{Bb}	16.2 ± 0.7^{Aa}	4.3 ± 0.5^{Ab}	4.4 ± 0.2^{ABb}	A x B	*<0.0001*
Water Holding Capacity (%)	1	96.4 ± 0.4^{Cb}	94.3 ± 0.9^{Da}	97.9 ± 0.3^{Bb}	97.2 ± 0.4^{Bb}		
	7	95.0 ± 0.3^{Cb}	91.1 ± 1.8^{Ca}	95.9 ± 0.7^{Ab}	94.5 ± 0.3^{Ab}	Sweetener (A)	*<0.0001*
	14	90.5 ± 0.7^{Ab}	86.0 ± 1.0^{Ba}	95.1 ± 1.5^{Ac}	96.9 ± 0.8^{Bc}	Time (B)	*<0.0001*
	21	94.3 ± 0.5^{Bb}	83.8 ± 0.7^{Aa}	95.7 ± 0.5^{Ab}	95.6 ± 0.2^{Bb}	A x B	*<0.0001*

Table S7. Rheological parameters (mean ± standard deviation) of the low-fat, low-protein yoghurts produced with different sweeteners. Different capital letters within the same column represent statistical differences between storage time for each parameter in the same sweetener. Different small letters within the same row represent statistical differences between sweeteners for each parameter in the same storage time ($p < 0.05$).

Parameter	Storage time (days)	Sucrose 6%	Stevia 0.03%	Agave 4.5%	*P-value*	
G' (Pa)	7	231.9 ± 37.4^{Bb}	366.9 ± 32.5^{Ba}	293.9 ± 8.5^{Bb}	Sweetener (A)	*<0.0001*
	14	265.7 ± 44.0^{Ac}	460.3 ± 54.2^{ABa}	368.4 ± 39.6^{Ab}	Time (B)	*<0.0001*
	21	370.7 ± 6.8^{Ab}	504.2 ± 29.4^{Aa}	411.2 ± 23.0^{Ab}	A x B	0.4287
G'' (Pa)	7	71.2 ± 10.6^{Bb}	106.1 ± 8.2^{Ba}	87.5 ± 1.8^{Bab}	Sweetener (A)	*<0.0001*
	14	78.3 ± 12.9^{Bc}	128.0 ± 13.8^{Aa}	105.3 ± 12.5^{ABb}	Time (B)	*<0.0001*
	21	106.1 ± 1.7^{Ab}	134.8 ± 9.4^{Aa}	115.2 ± 6.2^{Aab}	A x B	0.3866
η* (Pas)	7	38.6 ± 6.2^{Bb}	60.8 ± 5.3^{Ba}	48.8 ± 1.4^{Bb}	Sweetener (A)	*<0.0001*
	14	44.1 ± 7.3^{Bc}	76.0 ± 8.9^{Aa}	61.0 ± 6.6^{Ab}	Time (B)	*<0.0001*
	21	61.4 ± 1.1^{Ab}	83.1 ± 4.9^{Aa}	68.0 ± 3.8^{Ab}	A x B	0.4246
tan δ	7	0.307 ± 0.006^{Aa}	0.289 ± 0.004^{Ac}	0.298 ± 0.002^{Ab}	Sweetener (A)	*<0.0001*
	14	0.295 ± 0.003^{Ba}	0.278 ± 0.003^{Bb}	0.286 ± 0.003^{Bb}	Time (B)	*<0.0001*
	21	0.286 ± 0.004^{Ca}	0.267 ± 0.003^{Cb}	0.280 ± 0.003^{Ba}	A x B	0.6849

Table S8. Rheological parameters (mean ± standard deviation) of the low-fat, high-protein yoghurts produced with different sweeteners. Different capital letters within the same column represent statistical differences between storage time for each parameter in the same sweetener. Different small letters within the same row represent statistical differences between sweeteners for each parameter in the same storage time ($p < 0.05$).

Parameter	Storage time (days)	Sucrose 6%	Stevia 0.04%	Xylitol 6%	Honey 6%	*P-value*	
G' (Pa)	7	410.2 ± 195.4^{Aa}	808.2 ± 178.3^{Aa}	619.3 ± 123.8^{Aa}	665.9 ± 81.3^{Aa}	Sweetener (A)	0.1957
	14	825.0 ± 294.6^{ABa}	1014.4 ± 343.3^{Aa}	871.4 ± 125.2^{Aa}	726.7 ± 172.4^{Aa}	Time (B)	*0.0009*
	21	976.8 ± 177.7^{Aa}	1033.3 ± 121.8^{Aa}	981.6 ± 153.4^{Aa}	973.4 ± 324.7^{Aa}	A x B	0.7829
G'' (Pa)	7	120.8 ± 55.3^{Aa}	224.0 ± 46.7^{Aa}	181.8 ± 37.5^{Aa}	195.2 ± 23.1^{Aa}	Sweetener (A)	0.3056
	14	235.5 ± 80.6^{ABa}	276.2 ± 92.4^{Aa}	244.8 ± 30.6^{Aa}	201.7 ± 45.8^{Aa}	Time (B)	*0.0022*
	21	267.6 ± 46.9^{Aa}	276.7 ± 35.5^{Aa}	269.9 ± 42.3^{Aa}	266.9 ± 86.7^{Aa}	A x B	0.7333
η* (Pas)	7	68.0 ± 32.3^{Ba}	133.5 ± 29.3^{Aa}	102.7 ± 20.6^{Aa}	110.5 ± 13.4^{Aa}	Sweetener (A)	0.202
	14	136.5 ± 48.6^{ABa}	167.3 ± 56.6^{Aa}	144.1 ± 20.5^{Aa}	155.0 ± 36.7^{Aa}	Time (B)	*0.0009*
	21	161.2 ± 29.2^{Aa}	170.3 ± 20.2^{Aa}	162.0 ± 25.3^{Aa}	160.6 ± 53.5^{Aa}	A x B	0.7796
tan δ	7	0.297 ± 0.008^{Aa}	0.278 ± 0.004^{Ab}	0.293 ± 0.003^{Aa}	0.293 ± 0.004^{Aa}	Sweetener (A)	*<0.0001*
	14	0.287 ± 0.006^{Ba}	0.273 ± 0.001^{ABb}	0.281 ± 0.005^{Bab}	0.278 ± 0.003^{Bab}	Time (B)	*<0.0001*
	21	0.274 ± 0.003^{Ca}	0.268 ± 0.003^{Ba}	0.275 ± 0.002^{Ba}	0.275 ± 0.004^{Ba}	A x B	0.2737

Table S9. Texture parameters (mean ± standard deviation) of the low-fat, low-protein yoghurts produced with different sweeteners. Different capital letters within the same column represent statistical differences between storage time for each parameter in the same sweetener. Different small letters within the same row represent statistical differences between sweeteners for each parameter in the same storage time ($p < 0.05$).

Parameter	Storage time (days)	Sucrose 6%	Stevia 0.03%	Agave 4.5%	*P-value*	
**Hardness (g)**	1	13.9 ± 1.1^{Ca}	14.4 ± 0.2^{Ca}	13.1 ± 0.3^{Ba}	Sweetener (A)	*<0.0001*
	7	15.7 ± 0.9^{Bb}	17.1 ± 0.4^{Ba}	15.8 ± 0.3^{Aab}	Time (B)	*<0.0001*
	14	16.9 ± 0.8^{Bab}	17.9 ± 0.9^{ABa}	16.1 ± 0.5^{Ab}	A x B	0.1572
	21	19.2 ± 2.2^{Aa}	18.9 ± 0.8^{Aa}	17.2 ± 0.7^{Ab}		
**Adhesiveness (g.s)**	1	-19.1 ± 1.3^{Db}	-20.9 ± 0.7^{Dc}	-16.9 ± 0.9^{Da}	Sweetener (A)	*<0.0001*
	7	-7.8 ± 0.9^{Ba}	-9.1 ± 1.4^{Ba}	-8.1 ± 1.1^{Ba}	Time (B)	*<0.0001*
	14	-11.6 ± 0.5^{Ca}	-11.8 ± 1.4^{Ca}	-11.1 ± 0.4^{Ca}	A x B	*0.0009*
	21	-4.4 ± 0.6^{Aa}	-4.3 ± 0.4^{Aa}	-4.1 ± 0.5^{Aa}		
**Springiness**	1	0.95 ± 0.01^{Bb}	0.94 ± 0.04^{Bb}	0.98 ± 0.01^{Aa}	Sweetener (A)	*0.0015*
	7	0.94 ± 0.01^{Bb}	0.96 ± 0.03^{ABb}	0.99 ± 0.00^{Aa}	Time (B)	*<0.0001*
	14	0.99 ± 0.02^{Aa}	0.99 ± 0.00^{Aa}	0.99 ± 0.00^{Aa}	A x B	*0.0072*
	21	0.25 ± 0.02^{Ca}	0.24 ± 0.01^{Ca}	0.24 ± 0.01^{Ba}		
**Gumminess (g)**	1	7.3 ± 0.5^{Ca}	7.4 ± 0.2^{Ca}	6.6 ± 0.2^{Ba}	Sweetener (A)	*<0.0001*
	7	8.2 ± 0.6^{Bb}	9.1 ± 0.3^{Ba}	8.5 ± 0.3^{Aab}	Time (B)	*<0.0001*
	14	9.5 ± 0.3^{Aa}	9.6 ± 0.7^{ABa}	8.7 ± 0.2^{Ab}	A x B	0.0969
	21	10.1 ± 1.3^{Aa}	9.9 ± 0.5^{Aa}	9.0 ± 0.4^{Ab}		
**Cohesiveness**	1	0.53 ± 0.03^{ABa}	0.51 ± 0.01^{Aa}	0.51 ± 0.02^{Ba}	Sweetener (A)	0.5552
	7	0.52 ± 0.01^{Ba}	0.53 ± 0.01^{Aa}	0.54 ± 0.01^{Aa}	Time (B)	*0.0001*
	14	0.55 ± 0.01^{Aa}	0.54 ± 0.01^{Aa}	0.54 ± 0.01^{Aa}	A x B	0.1531
	21	0.52 ± 0.02^{Ba}	0.53 ± 0.03^{Aa}	0.53 ± 0.01^{ABa}		
**Resilience**	1	0.10 ± 0.01^{BCa}	0.11 ± 0.01^{Aa}	0.11 ± 0.02^{Ba}	Sweetener (A)	0.1981
	7	0.12 ± 0.01^{Ba}	0.10 ± 0.01^{Aa}	0.11 ± 0.01^{Ba}	Time (B)	*<0.0001*
	14	0.10 ± 0.00^{Ca}	0.11 ± 0.01^{Aa}	0.10 ± 0.00^{Ba}	A x B	*0.0080*
	21	0.14 ± 0.02^{Aa}	0.12 ± 0.01^{Ab}	0.13 ± 0.01^{Aa}		

Table S10. Texture parameters (mean ± standard deviation) of the low-fat, high-protein yoghurts produced with different sweeteners. Different capital letters within the same column represent statistical differences between storage time for each parameter in the same sweetener. Different small letters within the same row represent statistical differences between sweeteners for each parameter in the same storage time ($p < 0.05$).

Parameter	Storage time (days)	Sucrose 6%	Stevia 0.04%	Xylitol 6%	Honey 6%	*P-value*	
**Hardness (g)**	1	33.8 ± 2.0^{Cb}	42.0 ± 1.6^{Ba}	41.8 ± 1.8^{Ca}	35.2 ± 1.1^{Cb}	Sweetener (A)	*<0.0001*
	7	40.5 ± 1.2^{Ab}	43.6 ± 1.7^{Ba}	44.7 ± 0.6^{Ba}	38.3 ± 1.3^{Bb}	Time (B)	*<0.0001*
	14	40.5 ± 2.0^{Ab}	47.2 ± 0.9^{Aa}	48.3 ± 3.4^{Aa}	40.7 ± 1.3^{ABb}	A x B	*0.0124*
	21	43.0 ± 1.2^{Bb}	47.1 ± 1.0^{Aa}	49.2 ± 1.0^{Aa}	42.5 ± 1.8^{Ab}		
**Adhesiveness (g.s)**	1	-3.6 ± 0.7^{Aa}	-22.2 ± 2.0^{Ab}	-28.9 ± 0.9^{Bc}	-22.8 ± 1.7^{Bb}	Sweetener (A)	*<0.0001*
	7	-25.8 ± 0.8^{BCbc}	-27.3 ± 1.7^{Bc}	-20.1 ± 1.2^{Da}	-25.3 ± 1.7^{Cb}	Time (B)	*<0.0001*
	14	-24.9 ± 1.3^{Ba}	-29.4 ± 1.1^{Cb}	-33.2 ± 1.1^{Cc}	-24.0 ± 1.6^{BCa}	A x B	*<0.0001*
	21	-27.6 ± 1.6^{Cc}	-22.0 ± 1.0^{Ab}	-22.2 ± 0.9^{Ab}	-18.6 ± 0.3^{Aa}		
**Springiness**	1	0.95 ± 0.00^{Ac}	0.99 ± 0.00^{Aa}	0.98 ± 0.02^{Aab}	0.96 ± 0.02^{Abc}	Sweetener (A)	*<0.0001*
	7	0.96 ± 0.01^{Aa}	0.96 ± 0.02^{Ba}	0.97 ± 0.03^{Aa}	0.96 ± 0.01^{Aa}	Time (B)	0.1194
	14	0.96 ± 0.01^{Aab}	0.95 ± 0.01^{Bb}	0.97 ± 0.02^{Aab}	0.98 ± 0.02^{Aa}	A x B	*0.0005*
	21	0.96 ± 0.01^{Ab}	0.99 ± 0.01^{Aa}	0.98 ± 0.02^{Aab}	0.96 ± 0.02^{Ab}		
**Gumminess (g)**	1	20.1 ± 1.5^{Bb}	24.4 ± 2.2^{Aa}	23.3 ± 1.8^{Ba}	18.9 ± 1.3^{Bb}	Sweetener (A)	*<0.0001*
	7	21.7 ± 1.0^{ABab}	23.8 ± 2.3^{Aa}	22.8 ± 2.1^{Ba}	20.6 ± 1.0^{ABb}	Time (B)	*<0.0001*
	14	22.9 ± 1.4^{Abc}	24.2 ± 1.0^{Aab}	26.2 ± 1.1^{Aa}	21.6 ± 0.7^{Ac}	A x B	0.0584
	21	23.1 ± 0.7^{Ab}	25.2 ± 0.6^{Aa}	25.8 ± 0.6^{Aa}	21.8 ± 1.1^{Ab}		
**Cohesiveness**	1	0.60 ± 0.05^{Aa}	0.54 ± 0.01^{Ab}	0.56 ± 0.02^{Aab}	0.55 ± 0.01^{Ab}	Sweetener (A)	*0.0110*
	7	0.54 ± 0.01^{Ba}	0.55 ± 0.05^{Aa}	0.51 ± 0.04^{Ba}	0.54 ± 0.01^{Aa}	Time (B)	*0.0035*
	14	0.57 ± 0.05^{ABa}	0.51 ± 0.02^{Ab}	0.53 ± 0.02^{ABab}	0.53 ± 0.02^{Aab}	A x B	0.0660
	21	0.54 ± 0.01^{Ba}	0.54 ± 0.01^{Aa}	0.52 ± 0.02^{ABa}	0.55 ± 0.02^{Aa}		
**Resilience**	1	0.10 ± 0.01^{Aa}	0.10 ± 0.00^{Aa}	0.10 ± 0.01^{Aa}	0.09 ± 0.01^{Aa}	Sweetener (A)	*0.0041*
	7	0.09 ± 0.00^{Aa}	0.09 ± 0.00^{Ba}	0.09 ± 0.01^{Ba}	0.09 ± 0.01^{Aa}	Time (B)	*0.0002*
	14	0.09 ± 0.01^{Aa}	0.09 ± 0.00^{ABa}	0.09 ± 0.01^{ABa}	0.09 ± 0.01^{Aa}	A x B	0.2033
	21	0.09 ± 0.01^{Aab}	0.09 ± 0.01^{ABa}	0.09 ± 0.01^{ABab}	0.08 ± 0.01^{Ab}		

Table S11. Sensorial analysis results (mean ± standard deviation) regarding flavor, taste, consistency, and appearance of the yoghurts produced with low-protein (LP) and high-protein (HP) and different sweeteners. Different small letters within the same row represent statistical differences between sweetened yoghurts for each parameter ($p < 0.05$).

Parameter	Sucrose 6% (LP)	Stevia 0.03% (LP)	Agave 4.5% (LP)	Sucrose 6% (HP)	Stevia 0.04% (HP)	Xylitol 6% (HP)	Honey 6% (HP)	*P-value*
**Flavor**	6.6 ± 1.2^{ab}	7.0 ± 1.5^{a}	5.8 ± 1.4^{b}	6.9 ± 1.4^{a}	6.7 ± 1.7^{ab}	6.6 ± 1.2^{ab}	7.0 ± 1.3^{a}	*0.0200*
**Taste**	5.8 ± 1.4^{de}	8.0 ± 0.9^{a}	5.1 ± 1.7^{e}	7.5 ± 0.9^{ab}	6.4 ± 1.5^{cd}	6.9 ± 1.2^{bc}	6.8 ± 1.4^{bcd}	*<0.0001*
**Consistency**	6.1 ± 1.5^{c}	6.7 ± 1.8^{bc}	6.5 ± 1.5^{c}	8.0 ± 1.0^{a}	7.6 ± 1.5^{ab}	7.8 ± 1.1^{a}	7.7 ± 1.4^{ab}	*<0.0001*
**Appearance**	6.8 ± 1.6^{b}	7.0 ± 1.5^{b}	6.9 ± 1.4^{b}	8.0 ± 0.8^{a}	7.8 ± 1.3^{ab}	8.1 ± 0.8^{a}	7.8 ± 1.6^{ab}	*<0.0001*
